# Lupus vulgaris with a pseudo tumoral presentation, case report

**DOI:** 10.1093/omcr/omaf154

**Published:** 2025-09-15

**Authors:** Fatima Zohra El Ali, Salim Gallouj, Kaoutar Benchakroune, Imane Talhaoui, Ouiame El Jouari

**Affiliations:** Department of Dermatology, Mohamed VI University Hospital Center of Tangier, 90000, Morocco; Department of Dermatology, Mohamed VI University Hospital Center of Tangier, 90000, Morocco; Department of Dermatology, Mohamed VI University Hospital Center of Tangier, 90000, Morocco; Department of Dermatology, Mohamed VI University Hospital Center of Tangier, 90000, Morocco; Department of Dermatology, Mohamed VI University Hospital Center of Tangier, 90000, Morocco

**Keywords:** cutaneous tuberculosis, lupus vulgaris, infectious disease

## Abstract

Lupus vulgaris (LV) is a chronic, paucibacillary form of cutaneous tuberculosis. These often-mutilating lesions present a significant diagnostic challenge. We report the case of a 61-year-old patient who presented with a voluminous telangiectatic and ulcerated pseudo-tumoral mass measuring 10 cm in diameter, with a mutilating aspect. Histopathology of the skin lesion revealed granulomatous dermo-hypo dermatitis. Lymph node biopsy showed granulomatous inflammation. QuantiFERON-TB assay was positive, and the established diagnosis was pseudo-tumoral lupus vulgaris. The patient was successfully treated with standard four-drug anti-tuberculosis therapy.

This case highlights the importance of a multidisciplinary approach, integrating rigorous clinical, biological, and histopathological correlation for early diagnosis. Appropriate anti-tubercular treatment led to significant and rapid lesion improvement, preventing severe and mutilating complications.

## Introduction

Lupus vulgaris (LV) is a form of paucibacillary cutaneous tuberculosis characterized by chronic granulomatous lesions that can progress into extensive and severe forms, particularly on the face. These lesions are often disfiguring, causing significant aesthetic and functional impairment. Clinical diagnosis is often complicated by the morphological diversity of the lesions, necessitating a precise differential diagnosis [1]. Despite the availability of treatments, controlling lupus vulgaris remains complex due to diagnostic challenges, the risk of multidrug-resistant tuberculosis, and the frequent need for trial antituberculosis treatment in the absence of mycobacterial isolation. we report a case of a patient with cutaneous tuberculosis of the lupus vulgaris type, presenting as mutilating pseudo-tumoral form.

## Case report

**Figure 1:**
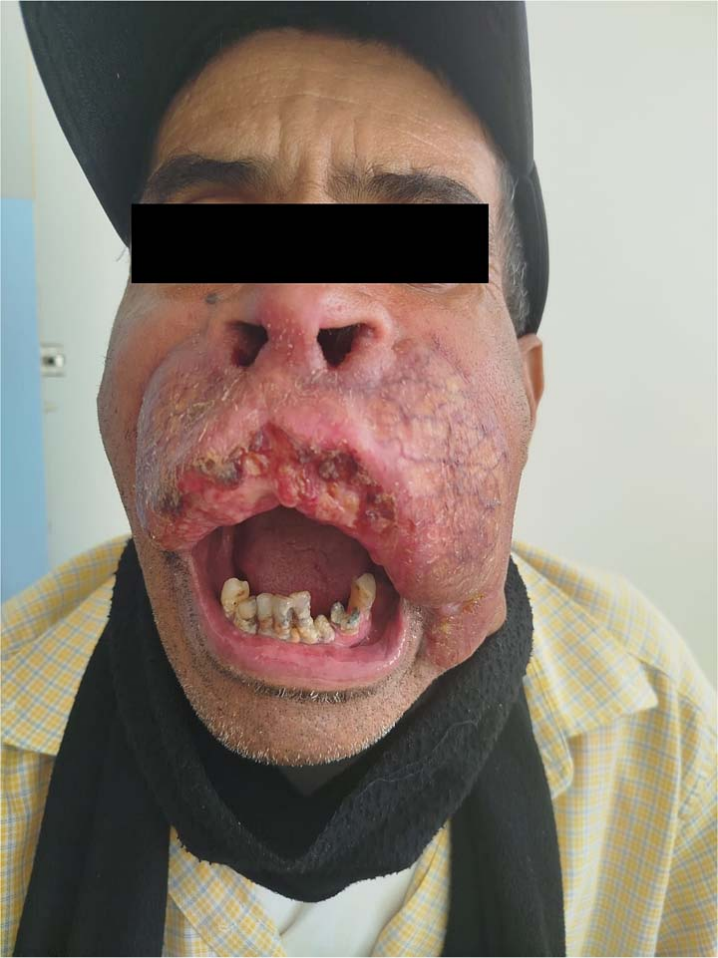
Initial clinical appearance showing a pseudo-tumoral lesion on the face, anterior view.

**Figure 2:**
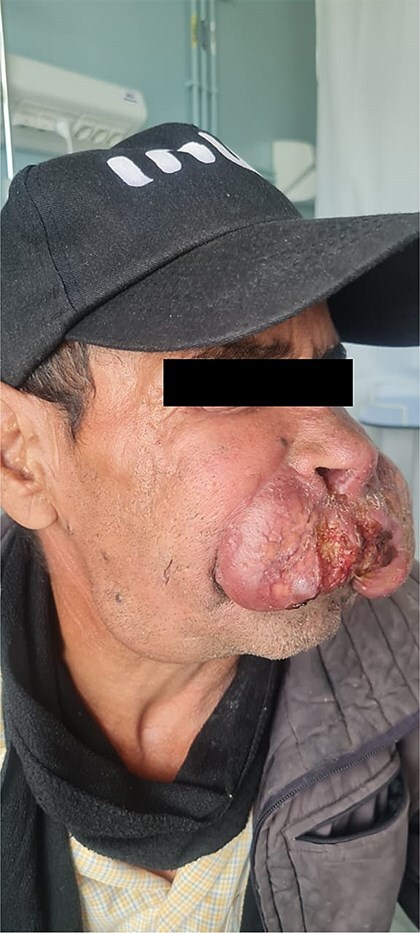
Initial clinical appearance showing a pseudo-tumoral lesion on the face, lateral view.

**Figure 3:**
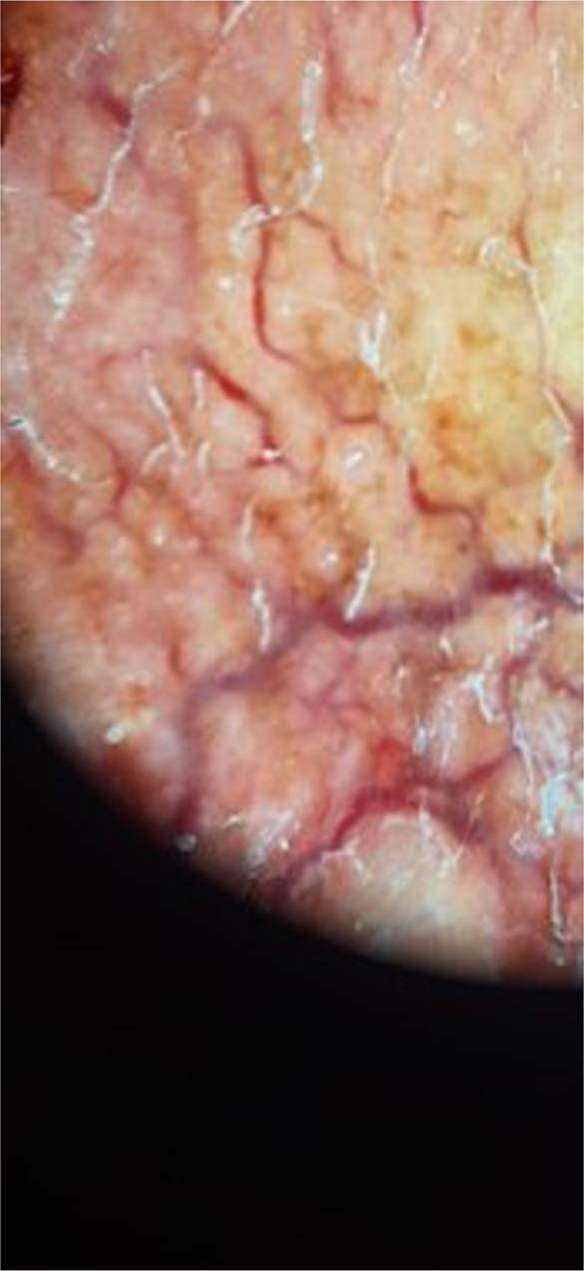
Dermoscopy findings showing erythematous background, structureless yellow-orange areas, arborizing linear vessels, fine whitish scales, and telangiectasias.

**Figure 4:**
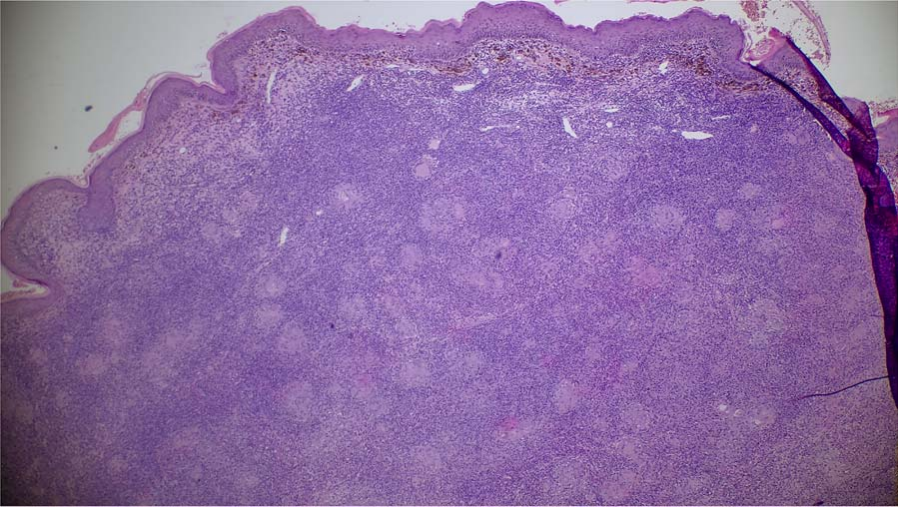
Image showing the histopathological findings on skin biopsy haematoxylin and eosin (H&E) stain x10 magnification.

**Figure 5:**
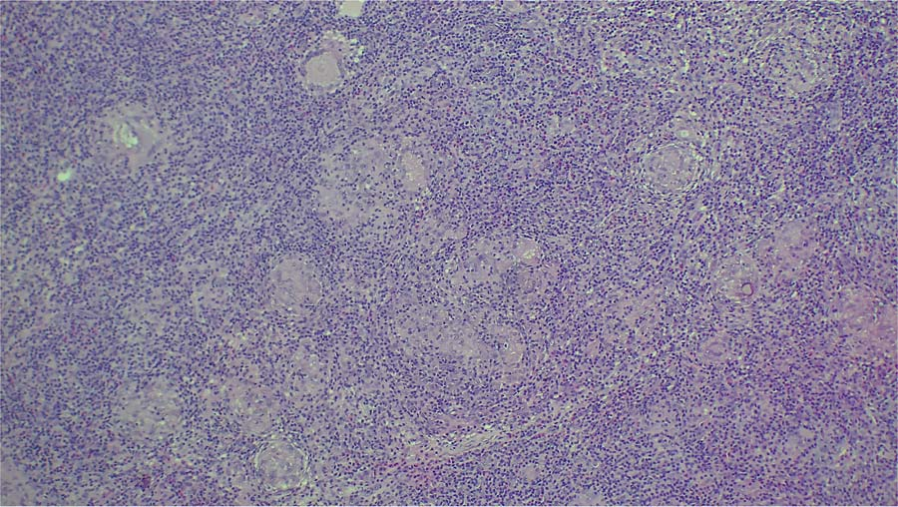
Image showing the histopathological findings on skin biopsy haematoxylin and eosin stain (H&E) x20 magnification.

**Figure 6:**
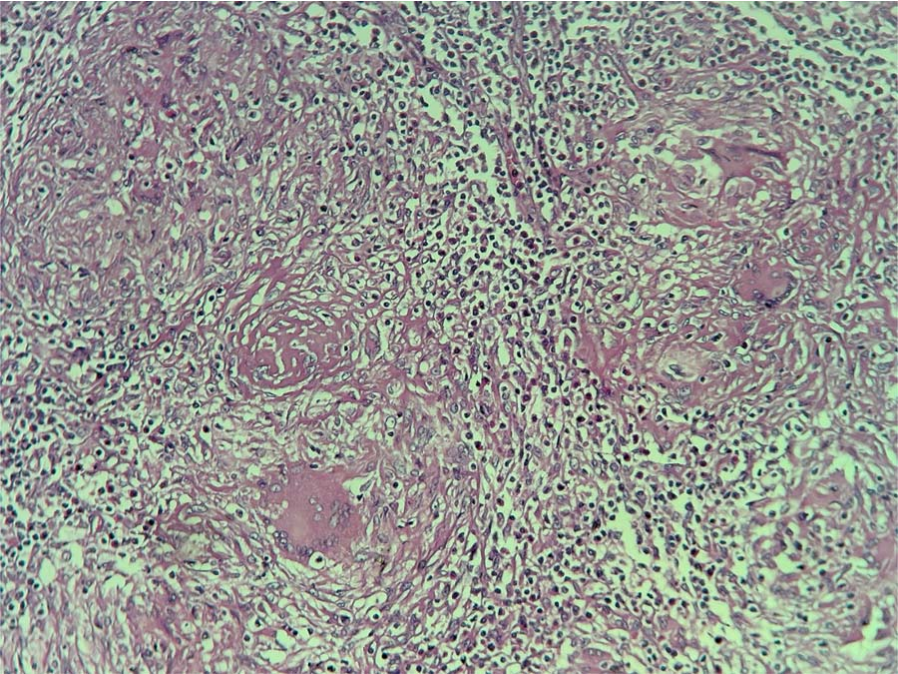
Image showing the histopathological findings of the lymph node biopsy, haematoxylin and eosin stain (H&E) x 10 magnification.

The patient was a 61-year-old immunocompetent farmer. He had no history of pulmonary tuberculosis or close contact with a tuberculosis case. He was a former smoker, having quit 8 years prior. The disease began a year earlier with the appearance of a painless, non-pruritic erythematous nodular lesion on the upper lip. The lesion gradually increased in size and changed appearance, becoming exophytic and ulcerative, without spontaneous bleeding or pus discharge. The evolution occurred in an afebrile context with preserved general health. Clinical examination revealed an ulcerated, telangiectatic, mutilating, and protruding tumour covering the entire upper lip, extending superiorly to the nasal root and laterally to the nasolabial, medial cheek, and mandibular regions, measuring 10 cm in its largest diameter. The lesion was centred by an ulcerated area covered with haemorrhagic and meliceric crusts, had a firm consistency, and was surrounded by apparently normal skin (Figs 1 and 2). Dermoscopic examination showed an erythematous background, structureless yellow-orange areas, arborizing linear vessels, fine whitish scales, and telangiectasias (Fig. 3). Oral mucosal examination revealed erosions on the upper lip and an inflamed palate. Lymph node examination identified bilateral, mobile, non-inflammatory cervical and mandibular lymphadenopathy. Histopathology of the skin lesion showed granulomatous dermo-hypo dermatitis without caseous necrosis, suggestive of an infectious origin, with no signs of malignancy (Figs 4, 5). Periodic acid–Schiff and Grocott’s methenamine silver stains were negative. Ziehl-Neelsen staining did not reveal acid-fast bacilli. Tissue cultures for mycobacteria and fungi were negative. The polymerase chain reaction test for tuberculosis was inconclusive, likely due to formalin fixation of the tissue. Lymph node biopsy revealed granulomatous inflammation (Fig. 6). QuantiFERON-TB assay was positive. GeneXpert MTB test on sputum to rule out active pulmonary tuberculosis was negative. Smear for leishmaniasis was negative. Complete blood count showed lymphopenia at 640/mm^3^ (normal: 1000–4800 cells/mm^3^).

Angiotensin-converting enzyme assay was negative. Liver and kidney function tests, and C-reactive protein were within normal ranges. Human Immunodeficiency Virus, and syphilis serologies were negative.

CT scan showed an ulcerative, exophytic, and budding soft-tissue process affecting the entire upper lip, irregular and poorly defined, measuring 10 cm in its longest axis, extending to the nasal region and bilateral premaxillary soft tissues. There was cortical erosion and an irregular lytic aspect of the maxillary alveolar process. Bilateral cervical lymphadenopathy was observed. The thoracic section showed a small cylindrical bronchiectasis with thickened walls and micronodules, along with traction bronchiectasis and calcifications in the lung apex.

Given the epidemiological context of the region and the clinical, biological, and histopathological findings, a diagnosis of cutaneous tuberculosis, lupus vulgaris type, was established. Antitubercular treatment was initiated as follows:

Intensive phase (2 months): Rifampicin (10 mg/kg/day), Isoniazid (5 mg/kg/day), Pyrazinamide (25 mg/kg/day), and Ethambutol (15 mg/kg/day).

Continuation phase (4 months): Rifampicin and Isoniazid.

**Figure 7:**
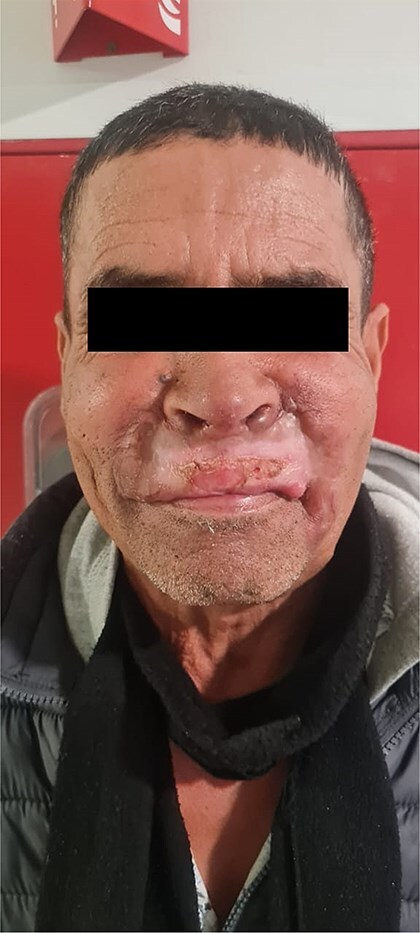
Clinical image showing clinical improvement of the patient after treatment, with volume reduction of tumour like lesion and of lymphadenopathy.

Clinical and biological monitoring was conducted every two months, including Complete Blood Count, liver and kidney function tests,the patient showed clinical improvement, with regression of the mass and inflammation, significant reduction in lymph node size, and disappearance of ulceration (Fig. 7).

## Discussion

Lupus vulgaris (LV) is a chronic paucibacillary form of cutaneous tuberculosis that typically affects individuals with prior sensitization and high immunity against *Mycobacterium tuberculosis* [1].this form is reported to be the most common in adults and the clinical presentation vary significantly, plaque-like, ulcerative, hypertrophic, vegetative, papular and nodular forms were described in literature [2] the pseudo-tumoral form is exceptionally reported [3, 4, 5]. Facial forms of lupus vulgaris, in particular, can evolve into ulcerative and exophytic mutilating lesions, complicating diagnosis and leading to therapeutic delays causing significant aesthetic and functional sequelae, as seen in our patient.

Histologically, lupus vulgaris is characterized by epithelioid granulomas located in the superficial dermis without caseous necrosis, with acid-fast bacilli rarely detectable in these granulomas [6]. Mycobacterial culture remains the gold standard but has a low positivity rate, making PCR a useful alternative for identifying *M. tuberculosis* [7]. Dermoscopy often complements these investigations, typically revealing linear vessels on a yellow-orange background [1], findings also observed in our patient.

Facial lupus vulgaris poses a diagnostic challenge due to its clinical resemblance to other dermatoses, such as mucocutaneous leishmaniasis, deep fungal infections, lepromatous leprosy, tertiary syphilis, and sarcoidosis [1]. Our investigations ruled out these diagnoses.

Despite the well-established efficacy of standard antitubercular treatments (isoniazid, rifampicin, pyrazinamide, and ethambutol), disease control remains problematic due to frequent diagnostic delays, the increasing emergence of drug-resistant *M. tuberculosis* strains, and potential co-infection with HIV [1, 6]. However, our patient tested negative for HIV.

These challenges highlight the importance of comprehensive and in-depth examination to detect potential systemic involvement, which is crucial for adapting therapeutic management and minimizing severe complications and mutilating sequelae [8, 9]. Infection occurs from a primary tuberculosis focus, which may be pulmonary, osteoarticular, lymphatic, or other organs. Correlating clinical presentation with advanced diagnostic strategies, including molecular testing and therapeutic trials, is essential for improving prognosis and optimizing disease control [1, 10].

Our case underscores the importance of early recognition of facial lupus vulgaris, particularly in its pseudo-tumoral form, a rare but severe manifestation of paucibacillary cutaneous tuberculosis. Diagnosis remains complex due to polymorphic presentations, microbiological challenges, and a wide range of differential diagnoses. A thorough clinicopathological and biological correlation enables early and accurate diagnosis. Properly administered antitubercular treatment leads to significant lesion improvement, limiting mutilating sequelae and enhancing the patient’s quality of life.
